# Supplementary material for: Comprehensive Annotation and Functional Exploration of MicroRNAs in Lettuce
Source: Front Plant Sci. 2021 Dec 24;12:781836. doi: 10.3389/fpls.2021.781836 (PMC8739914; doi:10.3389/fpls.2021.781836)
Supplement: Supplementary file 3 [file Data_Sheet_9.ZIP › GSM2230753.plot/Lsa-miRN1652a_Lsat_1_v5_gn_2_13161.1_131_TPlot.pdf]

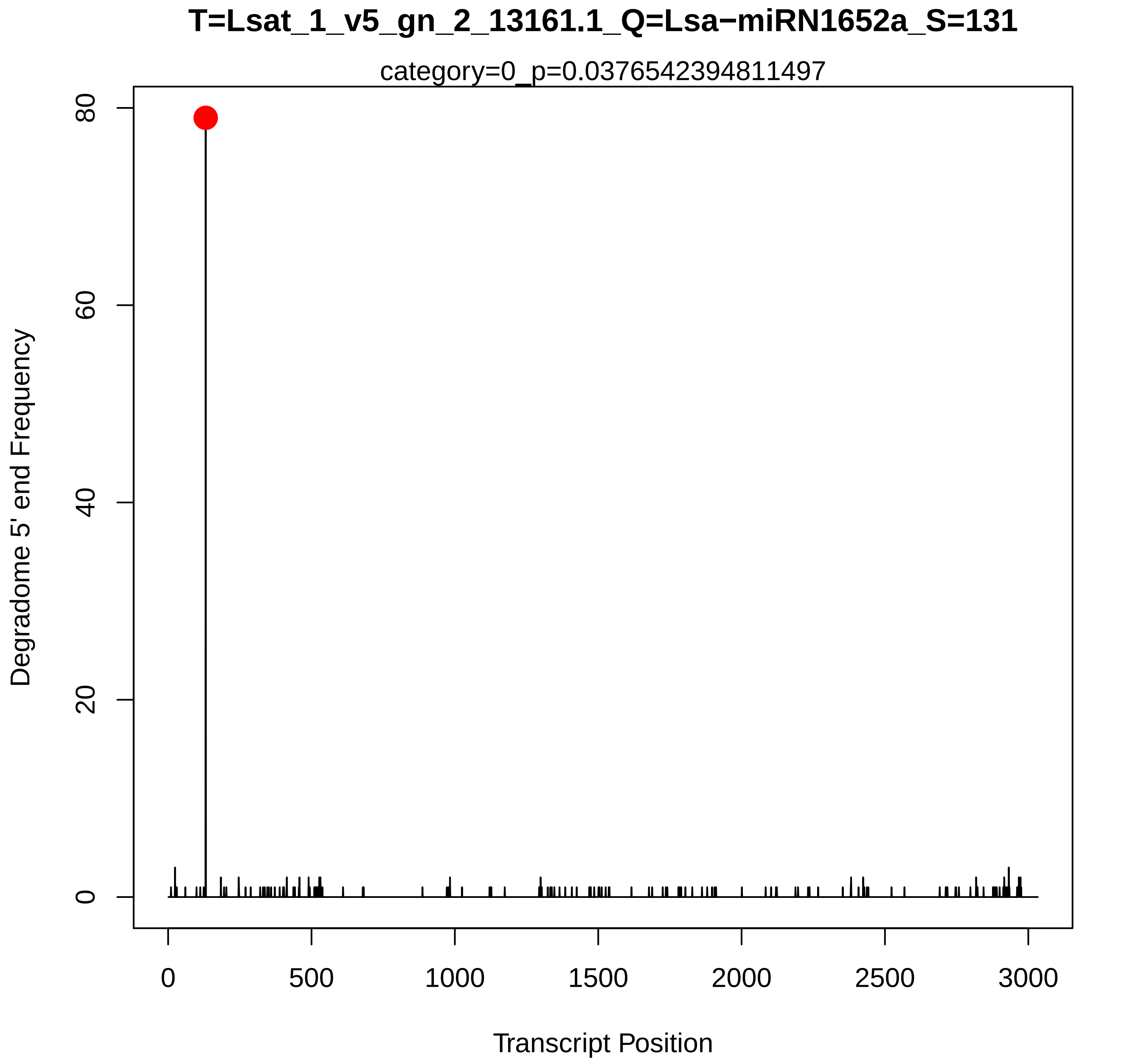
T=Lsat_1_v5_gn_2_13161.1_Q=Lsa−miRN1652a_S=131
category=0_p=0.0376542394811497
Degradome 5' end Frequency
Transcript Position
0
20
40
60
80
0
500
1000
1500
2000
2500
3000
